# Need for sustainable health policies toward curbing future pandemics in Africa

**DOI:** 10.1016/j.amsu.2022.104506

**Published:** 2022-08-30

**Authors:** Taiwo Oluwaseun Sokunbi, Jeremiah Oluwamayowa Omojuyigbe

**Affiliations:** Obafemi Awolowo University, Ile-Ife, 220101, Nigeria

Dear Editor,

The purpose of this paper is to highlight the need for long-term health policies to prevent future pandemics in Africa. A pandemic is the unplanned, global spread of an infectious disease or endemic that usually kills hundreds of thousands of people. Throughout history, at least four pandemic outbreaks have occurred, ranging from the plague to the current COVID-19 [1]. Human-to-human transmission is the most common method of spreading these pandemics due to our interaction with one another. As a result, several tactics and regulations were devised in an effort to halt disease transmission; these were designed to prevent or reduce human-to-human contact. To stop the spread of the disease, policies such as social gathering restrictions, quarantines, and patient isolation, among others, were implemented, as were border closures to limit travel within and outside of a country or city [1]. More than 7 million individuals worldwide have died from the COVID-19 pandemic alone, and despite the continent's limited testing capacity, roughly 253,000 of these victims were Africans. This is despite all the policies enacted to avoid lives lost in pandemics [2].

Some of the policies implemented during pandemics to control disease spread make life difficult for people, particularly in Africa. The majority of African households' live hand to mouth. As well-intended as public health policies such as movement restrictions and lockdowns to combat pandemics are, they put more people at risk of poverty and hunger. People would not want to stay indoors and die of hunger, so such policies would be disregarded [3]. In addition, if there is little compliance, government tactics and rules won't matter because the battle against the pandemic is already lost.

The COVID-19 pandemic showed the weaknesses in the health infrastructure and systems in African nations, which frequently make it challenging to reduce pandemic casualties. The present day's policies of lockdown, restriction of movement, sit-at-home, and social and physical distancing [3] pose more threats to the social and economic development of the continent. Due to their sociocultural heritage and level of economic development, other continents like Asia and Europe can adopt these strategies for battling pandemics. But because of their distinct socioeconomic and infrastructure issues, African nations put the sustainability of their citizens at risk by implementing these policies [3,4]. Therefore, there is a need for the development of suitable and sustainable health policies in African countries.

The healthcare system must be strengthened and revitalized to control abrupt exposure to disease epidemics without a sufficient structure [5]. It is worthy of note that the COVID-19 pandemic caught the world's health system by big shock following its emergence [6]. Global health systems, notably those in developing countries, are facing significant challenges as a result. The latest illness epidemics have had an impact on economies all across the world [5]. This, however, does not affect all in the same way or at the same time because each country's level of preparedness varies [5].

In Nigeria, public health experts and health care workers used lessons learned from the poliomyelitis response to combat the Ebola virus [6]. This emphasizes why a long-term health policy is required in the event of unprecedented pandemics in the future [6]. This Letter urges African policymakers, health experts, and governments to develop a long-term health policy to prepare for future pandemics.

Globally, systems are vulnerable to fragility complications ranging from health and wellness to food, employment, finance, and trade, and the consequences are still developing rapidly and remain unpredictable [5]. A lack of a developmental system will result in an epileptic health system in the event of an unexpected outbreak, such as the COVID-19 pandemic. This pandemic has exacerbated structural global health irregularities such as tribe, race, and access to health care. Priorities for these issues must be coordinated and expanded [7]. A disease outbreak can spread quickly between regions depending on the route of transmission - foreign travel, a lack of testing, or contact with an infected person. As a result, countries with limited preparedness find it difficult to control the spread, resulting in a larger problem.

Health policies can be related to past outbreaks. To develop a sustainable health policy, policy makers and concerned health care officials should be thinking about important questions and lessons. One step in this direction is to understand illness onset and history both within and beyond the country [5]. Another step is to continue to collaborate with health organizations such as the World Health Organization (WHO) to seek direction and support in research and surveillance.

When dealing with disease outbreaks, health policymakers and advisors should have access to scientific guidance and evidence-based research. Policy debates have focused on the need for stimulus programs to keep the economy running after the pandemic has passed [5]. While investments may only be able to fix the current structure, efforts should also be directed toward capturing prospects for economic growth in the post-pandemic era.

It is not too soon to begin thinking about Africa's healthcare policy in the event of a future pandemic. Certain outbreak scenarios have highlighted the importance of long-term health policy and strategy, as well as adequate data to guide and ensure good policymaking and prompt preparedness for future outbreaks.

## Ethical approval

Not applicable.

## Sources of funding

No funding received.

## Author contribution

All authors contributed to the writing of the paper, editing and final approval of the paper.

## Registration of research studies

Name of the registry:

Unique Identifying number or registration ID:

Hyperlink to your specific registration (must be publicly accessible and will be checked):

## Guarantor

Taiwo Oluwaseun Sokunbi.

## Declaration of competing interest

The authors declare that they have no known competing financial interests or personal relationships that could have appeared to influence the work reported in this paper.
